# Supplementation of *Lactobacillus plantarum* K68 and Fruit-Vegetable Ferment along with High Fat-Fructose Diet Attenuates Metabolic Syndrome in Rats with Insulin Resistance

**DOI:** 10.1155/2013/943020

**Published:** 2013-04-16

**Authors:** Hui-Yu Huang, Mallikarjuna Korivi, Chun-Han Tsai, Jo-Hsuan Yang, Ying-Chieh Tsai

**Affiliations:** ^1^Department of Food Science, Nutrition, and Nutraceutical Biotechnology, Shih-Chien University, Taipei City 10462, Taiwan; ^2^Division of Mental Health and Addiction Medicine, Institute of Population Health Sciences, National Health Research Institutes, Zhunan 35053, Taiwan; ^3^Department of Sports Sciences, TPEC, Taipei City 11153, Taiwan; ^4^Institute of Biochemistry and Molecular Biology, National Yang-Ming University, Taipei City 11221, Taiwan

## Abstract

*Lactobacillus plantarum* K68 (isolated from *fu-tsai*) and fruit-vegetable ferment (FVF) have been tested for antidiabetic, anti-inflammatory, and antioxidant properties in a rat model of insulin resistance, induced by chronic high fat-fructose diet. Fifty rats were equally assigned into control (CON), high fat-fructose diet (HFFD), HFFD plus K68, HFFD plus FVF, and HFFD plus both K68 and FVF (MIX) groups. Respective groups were orally administered with K68 (1 × 10^9^ CFU/0.5 mL) or FVF (180 mg/kg) or MIX for 8 weeks. We found that HFFD-induced increased bodyweights were prevented, and progressively increased fasting blood glucose and insulin levels were reversed (*P* < 0.01) by K68 and FVF treatments. Elevated glycated hemoglobin (HbA1c) and HOMA-IR values were controlled in supplemented groups. Furthermore, dyslipidemia, characterized by elevated total cholesterol (TC), triglyceride (TG), and low-density lipoproteins (LDLs) with HFFD, was significantly (*P* < 0.01) attenuated with MIX. Elevated pro-inflammatory cytokines, interleukin-1*β* (IL-1*β*), IL-6, and tumor necrosis factor-*α* (TNF-*α*), were controlled (*P* < 0.01) by K68, FVF, and MIX treatments. Moreover, decreased superoxide dismutase (SOD), catalase (CAT), and glutathione peroxidase (GPx) activities were substantially (*P* < 0.01) restored by all treatments. Experimental evidences demonstrate that K68 and FVF may be effective alternative medicine to prevent HFFD-induced hyperglycemia, hyperinsulinemia, and hyperlipidemia, possibly associated with anti-inflammatory and antioxidant efficacies.

## 1. Introduction

The major recent challenge in medical or food science in developed/developing counties is to combat against the diet related disorders, especially diseases connected to insulin resistance (IR) syndrome. IR represents a cluster of metabolic disorders, including obesity, glucose intolerance, and predisposes to type 2 diabetes [1, 2]. Occurrence of IR is mainly due to the urbanization and consequent changes in lifestyle, particularly, shifting the regular diet to “Western-style diet” which contains high fat [1, 3, 4]. Chronic high fat intake has been proved as a key responsible factor for metabolic syndrome [5]. In addition, high fructose consumption progresses to dietary model of type 2 diabetes that is associated with obesity, IR, hyperglycemia, and dyslipidemia [2]. Intake of either fat or fructose diet has been shown to trigger the free radicals or reactive oxygen species (ROS) production, thus ruining the antioxidant and inflammatory systems [5–7].

Many traditional ethnicities in East Asia and around the world believe that functional foods (fermented) are rich in nutrients and used as alternative medicine. Preserved foods have been used as condiments to enhance the overall flavor of the meal. Fermented food products contain several useful bacteria, including lactic acid bacteria (LAB), and easier to digest than unfermented foods [8]. Various strains of LAB are used in the manufacture of fermented foods, including milk, bread, vegetables, and other edible plant materials [9, 10]. Certain strains of LAB, particularly strains from the genera *Lactobacillus*, showed several health promoting effects, including antidiabetic property [11, 12]. Among several *Lactobacillus* species, *L. plantarum* is versatile species that possesses several therapeutic applications. *L. plantarum* 299v is reported as cardioprotective agent by decreasing the plasma insulin, leptin, and IL-6 concentrations in smokers [13]. However, only few studies demonstrated the applications of *L. plantarum* in diabetic rat model. In view of the global threat from obesity and diabetes, it is highly essential to treat/prevent the diet-induced metabolic syndrome by enhancing the intake of proper nutrients, which has been considered as alternative medicine.

Our previous findings demonstrated the diversity of LAB from various kinds of Taiwanese fermented foods, such as stinky tofu,* suan-tsai,* and *fu-tsai* [14, 15]. *L. plantarum* K68 (K68), a probiotic strain isolated from *fu-tsai*, has been shown to reduce the production of pro-inflammatory cytokines (TNF-*α*, IL-*β*, and IL-6) in dextran sulfate sodium (DSS)-induced ulcerative colitis BALB/c mice [16]. Frohlich et al. [17] reported the protective effect of fermented food substance against the development of cancer.

Since indigenous fermented fruits and vegetables tremendously contribute health promoting effects, it is necessary to validate the scientific facts of their ingredients. Therefore, we made an attempt to evaluate the medicinal values of  *L. plantarum* K68 and fruit-vegetable ferment (FVF) against high fat-fructose diet (HFFD)-induced metabolic syndrome in rats. In this study, we fed rats with HFFD to induce insulin resistance and simultaneously supplemented K68 and FVF to examine their therapeutic effects on the progression of obesity, hyperglycemia, hyperinsulinemia, and hyperlipidemia. To emphasize the significance of traditional food as a complementary and alternative medicine, we further investigated the anti-inflammatory and antioxidant properties of these substances in IR rats.

## 2. Materials and Methods

### 2.1. Preparation of *L. plantarum* K68 from Fermented *fu-tsai*

In this study, *L. plantarum* K68 was prepared as described in the previous studies [15, 16]. Briefly, K68 was prepared from the locally available fermented food, *fu-tsai*, and preserved in our LAB bank. For the experiments, K68 was inoculated in MRS broth and cultured at 30°C for 21 hrs. The isolated bacteria was harvested using centrifugation for 10 min at 1500 g, then washed twice with sterile PBS, and then resuspended to a final concentration of 1 × 10^9^ CFU/mL. The entire procedure was carried out under hygienic conditions at the Institute of Biochemistry and Molecular Biology, National Yang-Ming University. In our previous study, we used different doses of *K68* in cell culture and animal studies and demonstrated that oral administration of K68 is effective in reverting the DSS-induced ulcerative colitis in mice through the anti-inflammatory and immunomodulatory activities [16]. Based on these evidences, the dose 1 × 10^9^ CFU/0.5 mL of K68 was selected in the present study to evaluate the antidiabetic, antioxidant, and anti-inflammatory properties.

### 2.2. Preparation of Fruit and Vegetable Ferment (FVF)

Fruit and vegetable ferment (FVF) was obtained from the Fu Gui Bioscience Co. Ltd., Chayi City, Taiwan. FVF used in this study is commercially available food in Taiwan, which contained several fruits and vegetables, mainly available in Taiwan area. All fruits and vegetables were cut into small pieces and then preserved for one year at 25°C. The fruits in FVF include jujube, mini melon, apple, strawberry, grapefruit, orange, lemon, pears, pineapple, papaya, longan, litchi, mango, hami melon, watermelon, tomato, plum, mulberry, avocado, kiwi, coconut, guava, sugar cane, fig, banana, and passion fruit. The vegetables (herbs) are such as cucumber, pumpkin, *Benincasa hispida*, mushroom, eggplant, cabbage, spinach, seaweed, Chinese cabbage, water spinach, celery, *Crataegus pinnatifida, Artemisia capillaries, Houttuynia cordata, Polygonatum odoratum* (Mill.) Druce, *Anemarrhena asphodeloides*, *Eleutherococcus senticosus*, *Taxillus parasitica, Lophatherum gracile, *and* Ligustrum lucidum *Fructus. The detailed nutrition values and bioactive compounds existed in FVF were determined at Food Industry Research and Development Institute, Hsinchu, Taiwan, and the values were represented in Table 2. The final gummy-like substance was dissolved in water and dose equivalent to 180 mg/kg bodyweight was administered orally to rats. According to the calculations of US Food and Drug Administration (FDA), Center for Drug Evaluation and Research (CDER), July 2005 [18], the dose of FVF was calculated for average human bodyweight. The following equation was employed to convert the animal dose to human.

Human Equivalent Dose (mg/kg) = Animal dose (mg/kg) × Animal Km/Human Km (0.162).

## 3. Animal Care and Maintenance (*In Vivo*)

Fifty healthy male Sprague-Dawley rats weighing 155 ± 5 g were obtained from the National Laboratory of Animal Breeding and Research Center, Taipei, Taiwan. All the rats were housed in clean polypropylene cages and maintained in animal house with controlled temperature (23 ± 2°C) and alternating 12 h dark and 12 h light cycle. After one week acclimatization to laboratory conditions, all rats in experimental groups had free access to high fat-fructose diet, while control animals were fed a standard diet (AIN-93M, PMI Nutritional International LLC, Saint Louis, MO, USA) and water *ad libitum*. The entire study design and all experimental protocols used in this study followed the guidelines of Care and Use of Laboratory Animals, and the study was approved by the Animal Care and Ethics Committee of Shih-Chien University.

### 3.1. Preparation of High Fat-Fructose Diet (HFFD, High Calorie Diet)

The HFFD used in this study was modified from the standard diet AIN-93M formula. Briefly, the standard diet contained 10% sucrose and 4% fat which were raised to 28% and 22%, respectively, by adding the additional 18% sucrose and 18% animal oil (lard) to the sucrose and fat portions. In addition to that, 20% fructose was additionally added to the standard diet in order to get high energy diet that is, 141 Kcal/30 g, where the standard diet comprised 114 Kcal/30 g only. The detailed components in high fat-fructose (modified) diet were shown in Table 1. Food and water intakes were recorded daily. Food conversion efficiency (FCE) ratio was calculated accordingly (FCE = food intake (g)/weight gain (kg) × 10^2^). Change in bodyweight on every other day was also recorded for all groups throughout the study.

### 3.2. Grouping and Treatment

All the rats were assigned into five groups, ten in each, and treated as follows.

*Group I. Control (CON).* Ten rats in this group consumed the standard laboratory diet and served as healthy control. For equivalent handling to other experimental groups, 0.9% saline was orally administered to all rats instead of supplements.

*Group II. High Fat-Fructose Diet (HFFD).* Rats in this group (*n* = 10) consumed high fat-fructose diet for 8-week period and received saline solution similar to control.

*Group III. High Fat-Fructose Diet Plus K68 (HFFD + K68).* In addition to the high fat-fructose diet, rats in this group (*n* = 10) were supplemented with K68 for a period of 8 weeks at the dose of 1 × 10^9^ CFU /0.5 mL per day. K68 was provided orally via an orogastric tube.

*Group IV. High Fat-Fructose Diet Plus FVF (HFFD + FVF).* All rats in this group were fed on a high-fat-fructose diet and treated with FVF product for 8 weeks. Based on the* in vitro* studies, 180 mg/kg bodyweight was provided orally by an orogastric tube.

*Group V. High Fat-Fructose Diet Plus MIX (HFFD + MIX).* Rats in this group were fed a high-fat-fructose diet along with the mixture of both K68 plus FVF substances (MIX). The dose and rout of administration was similar to groups III and IV.

## 4. Biochemical Evaluations

### 4.1. Measurement of Fasting Blood Glucose, Serum Insulin, and HbA1c

On every week from week 0 to week 8, fasting blood samples were collected from the tail vein. Fasting blood glucose levels were determined immediately by using the glucose analyzer (LifeScan, Milpitas, CA, USA). Simultaneously, about 200 *μ*L blood sample was transferred into labeled centrifuge tubes and then centrifuged at 3500 rpm for 10 min to obtain serum for insulin assay. According to the protocol provided by Mercodia Insulin ELISA kit (Uppsala, Sweden), serum insulin levels were assayed. The endpoint was read spectrophotometrically on an ELISA plate reader at 450 nm (BioTek, PowerWave XS2, Vermont, USA). Both blood glucose and insulin levels were measured on every week.

Furthermore, the homeostasis model assessment of basal insulin resistance (HOMA-IR) was calculated at week 6 and week 8 by using fasting serum insulin and blood glucose levels according the following equation:

(1) $$\begin{array}{c} \text{HOMA}-\text{IR}\;\text{index}=\frac{\text{insulin}\left(\mu \text{U}/\text{mL}\right)\times \text{glucose}\left(\text{mmol}/\text{L}\right)}{22.5}. \end{array}$$

After 8-week treatment, a fraction of whole blood sample was used to detect the glycated hemoglobin (HbA1c) as described by the Randox HbA1c Assay Kit (Randox, Antrim, United Kingdom).

### 4.2. Oral Glucose Tolerance Test (OGTT)

Oral glucose tolerance test was performed after 6-week treatment in this study. All the rats were fasted for 12 h prior to test, and OGTT was conducted in the morning between 7.00 and 9.00 AM under fasting condition. One gram of glucose solution (50%, w/v) was orally administered to all rats before performing the test. Blood samples were collected from the tail vein by tail milking at 0 min (fasting sample) and 30, 60, 120, and 180 min time points after oral glucose administration (1 g/kg bodyweight). Glucose tolerance was determined by measuring the blood glucose and serum insulin concentrations for every 30 min interval. The glucose analyzer was used to determine the blood glucose levels (Lifescan, Milpitas, USA), and serum insulin levels were quantified on an ELISA analyzer as described in the previous section.

### 4.3. Assessment of Serum Lipid Profiles and Adipokines

Lipid profiles, including total cholesterol (TC), triglycerides (TG), low-density lipoprotein (LDL), and high-density lipoprotein (HDL) levels, were estimated in the serum of freshly collected blood sample. Prior (12 h) to the blood collection, the feed was removed from each cage, and samples were collected under fasting condition. TC, TG, and HDL levels were detected by commercial kits provided by the Randox (Randox Laboratories Limited, BT29 4QY, UK). LDL levels were calculated accordingly by using the Friedewald equation [19]. All the values were expressed as mg/dL.

Important adipokines, adiponectin, and leptin, which are associated with bodyweight changes, were determined in the serum. As per the Assaypro Rat Adiponectin ELISA kit's protocol, serum adiponectin levels were estimated (St. Charles, MO, USA). Circulating leptin levels were determined according to the protocol provided by RayBio rat leptin ELISA kit (Norcross, GA, USA). Absorbance of the sample was read immediately after adding the stop solution at 450 nm in a spectrophotometer (BioTek, PowerWave XS2, VT, USA). Adiponectin and leptin values were represented as ng/mL and pg/mL, respectively.

### 4.4. Determination of Circulating Pro-Inflammatory Cytokines

Circulating pro-inflammatory cytokines, including interleukin-1*α* (IL-1*α*), IL-6, and tumor necrosis factor-*α* (TNF-*α*), concentrations were evaluated by commercially available kits obtained from eBioscience (San Diego, CA, USA). The final absorbance for all cytokines (IL-1*α*, IL-6, and TNF-*α*) were monitored at 450 nm on an ELISA plate reader (BioTek, PowerWave XS2, Vermont, USA). The concentrations of all inflammatory markers were expressed as pg/mL of serum.

### 4.5. Assessment of Antioxidant Enzyme Activities

Three major antioxidant enzymes in serum including superoxide dismutase (SOD), catalase (CAT), and glutathione peroxidase (GPx) activities were assayed by using the Cayman assay kits (Anna Arbor, MI, USA). Serum SOD activity was determined on a ELISA plate reader. The final absorbance was read at 450 nm (Tecan Genios, A-5082, Austria), and activity was expressed as units/mL. CAT activity was measured by adding the H_2_O_2_ to the sample as per the protocol described in Cayman Catalase Assay Kit, and the activity was expressed nanomoles/mL/min. By using the NADPH, serum GPx activity was assayed. The reduction in the absorbance was read at 340 nm for every minute to get at least 5 time points by using a plate reader (Tecan Genios, A-5082, Austria). GPx activity was presented as units/mg protein. Protein concentrations in the samples were determined by Bio-Rad protein assay.

## 5. Assessment of FVF Antioxidant Capacities (*In Vitro*)

### 5.1. DPPH Scavenging Activity

2,2-Diphenyl-1-picrylhydrazyl (DPPH) radical scavenging activity was determined by the method of Liyana-Pathirana and Shahidi [20]. The absorbance of the mixture was measured spectrophotometrically at 517 nm. The ability of the FVF extract to scavenge DPPH radical was calculated by the equation

(2) DPPH  radical  scavenging  activity  =[Abs  control−Abs  sampleAbs  control]×100.

### 5.2. Superoxide Radical (O_2_^•−^) Scavenging Activity

Superoxide anion scavenging activity was measured by method described by Robak and Gryglewski [21]. O_2_^•−^ were generated in PMS-NADP system by oxidation of NADH and assayed by the reduction of nitro blue tetrazolium (NBT). Decreased absorbance of the reaction mixture indicates the increased superoxide anion scavenging activity. The percentage inhibition of superoxide anion generation was calculated using the following formula: superoxide anion scavenging activity (%) = [(A_cont_ − A_test_)/A_cont_] × 100, where A_cont_ was the absorbance of control reaction, and A_test_ was the absorbance of the extract or standards.

### 5.3. Angiotensin-Converting Enzyme (ACE) Activity

ACE activity was measured by using the substrate hippuryl-L-histidyl-L-leucine (HHL). ACE cleaves the substrate to expose to a free N-terminus, which could fluorogenically be labeled with o-phthaldialdehyde (OPA). Fluorescent was read at 355 nm excitation and 535 nm emission on a fluorescent spectrophotometer.

### 5.4. Total Phenol

The phenolic content present in FVF was determined spectrophotometrically by the Folin Ciocalteu modified method as described by Wolfe et al. [22]. Sample absorbance was measured spectrophotometrically at 765 nm (BioTek, PowerWave XS2, VT, USA). The measurements were conducted in triplicate. The obtained results were expressed as mg/g tannic acid equivalent from the calibration curve.

The vitamin E levels were evaluated by HPLC (Hitachi L2400), and selenium levels were estimated by atomic absorbance spectrophotometer (Perkin Elmer 5100). Both assays were performed at Food Industry Research and Development Institute, Hsinchu, Taiwan.

### 5.5. Statistical Analyses

The obtained data were analyzed by MS Office Excel and SPSS software. All the data were expressed by means ± SEM for ten replicates. The significance among the groups was achieved by two-way ANOVA along with Tukey's multiple-range post hoc test. The significance level was set as *P* < 0.05.

## 6. Results

### 6.1. Bioactive Ingredients in FVF

All active ingredients existing and responsible for the pharmacological effects of fermented fruit-vegetable (FVF) are estimated. In addition to proteins and carbohydrates, FVF is rich with several minerals, such as sodium (Na, 87.44 mg), potassium (K, 493 mg), calcium (Ca, 48.3 mg), magnesium (Mg, 39.4 mg), ferrous (Fe, 1.35 mg), and phosphorous (61 mg/100 g). Furthermore, FVF also possesses vitamin B-complex (B-1 (thiamin), B-2 (riboflavin), B-6, B-7 (biotin), and B-9 (folic acid)) and other antioxidants, including vitamin E and selenium (Se). The detailed nutrition values with bioactive compounds were listed in Table 2.

### 6.2. Confirmation of Insulin Resistance (IR)

Circulating insulin and glucose concentrations were regularly monitored for all groups under fasting condition. We found higher serum insulin levels (1.5 ng/dL) after 4 weeks along with elevated blood glucose concentrations in HFFD-fed rats (Figures 2(a) and 2(b)). The progressively increase in serum insulin (hyperinsulinemia) and blood glucose (hyperglycemia) levels with HFFD considered as insulin resistance in rats.

### 6.3. Impact of Chronic High Fat-Fructose Diet on Metabolic Parameters

**Figure 1:**
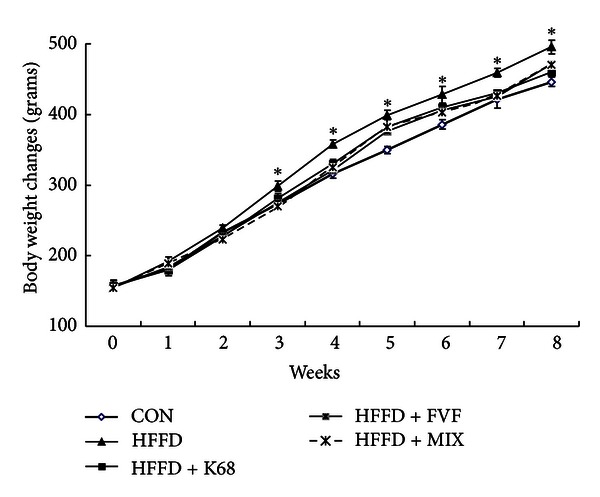
Changes in whole bodyweight over a period of 8 weeks with high fat-fructose diet, and K68, FVF, and MIX supplements in rats (*N* = 10). *: significant compared to CON group.

Collected data indicates that progressively increased whole bodyweight from week 0 to week 8 with high fat-fructose diet clearly reveals the negative impact of chronic HFFD intake in rats (Figure 1).  Table 4 shows that weight gain in HFFD group was 341.6 ± 5.73 g, where it was only 288.7 ± 1.6 g in control group that was fed a standard diet. The weight management effects of K68, FVF, and MIX supplements have been noticed from week 3 with a significant (*P* < 0.01) lower bodyweights compared to the untreated HFFD group. This trend was continued until week 8 (Figure 1).

Individual organ weights, including liver, kidney, and epididymal fat weights, were recorded at the end of the study. We found that liver weight was partially, and epididymal fat weight was significantly (*P* < 0.05), increased with HFFD compared to standard diet. However, these changes were controlled in all supplemented groups. Particularly, epididymal fat weight was significantly reduced. Although the average daily food intake was not significantly altered among the groups, FCE ratio was marginally decreased in HFFD and FVF as well as MIX supplemented groups compared to control group (Table 4).

### 6.4. Antihyperglycemic and Antihyperinsulinemic Effects of K68 and FVF

In order to determine the antidiabetic properties K68 and FVF, fasting blood glucose, insulin, and HbA1c concentrations were measured. Data clearly showed that intake of diet with high fat-fructose affects the circulating blood glucose and insulin concentrations. Elevated blood glucose and insulin levels at week 6 were 54% and 61.4%, respectively, and that was progressed to 75.5% and 110.7% at week 8 compared to the control diet group. Calculated HOMA-IR values were also significantly (*P* < 0.01) increased in HFFD group (3.28 ± 0.08) than CON group (1.32 ± 0.04) at week 6. Interestingly, supplementation of K68 and FVF to HFFD groups showed a trend in decreasing the blood glucose, insulin, and HOMA-IR values at week 6. This decrease trend was statistically significant with combination of K68 plus FVF (MIX) supplement compared to HFFD (Table 5).

**Figure 2:**
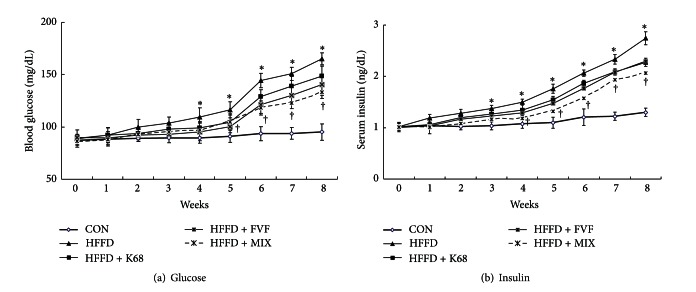
Changes in blood glucose (a) and insulin (b) levels over a period of 8 weeks with K68, FVF, and MIX supplements along with HFFD in rats (*N* = 10). *: significant compared to CON; ^†^: significant compared to HFFD group.

Statistical analyses showed that at week 8 blood glucose, insulin, HbA1c, and HOMA-IR values were recorded significantly (*P* < 0.01) lower with all supplements (K68, FVF, and MIX) (Table 5). Although supplements were unable to reduce those diabetes indicators completely, however, the treatment was effective to avoid the further progression of diabetic condition. This was further convinced by weekly monitored fasting blood glucose and insulin concentrations, which showed supplements setback of the diet-induced elevated glucose and insulin levels from week 4 till the end of the study (Figures 2(a) and 2(b)). 

**Figure 3:**
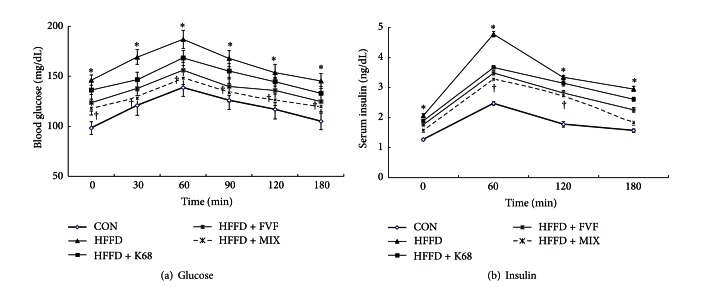
Effect of K68, FVF, and MIX supplementation on oral glucose tolerance test (OGTT) in rats fed a HFFD for 6 weeks (*N* = 10). *: significant compared to CON; ^†^: significant compared to HFFD group.

Furthermore, blood glucose and insulin levels under an oral glucose challenge (OGTT) were significantly (*P* < 0.01) higher at all time points in HFFD group and unable to reach baseline until 180 min. However, K68, FVF, and MIX supplementation to HFFD rats maintained the lower glucose and insulin levels against an oral glucose challenge (Figures 3(a) and 2(b)).

### 6.5. Effect of K68 and FVF on Dyslipidemia and Adipokines with HFFD

Estimated lipoprotein levels, including total TC, TG, LDL, and HDL, were represented in Table 6. As we expected, Tukey's post-hoc analyses showed that circulating TC, TG, and LDL levels were significantly (*P* < 0.01) elevated with HFFD compared to standard diet. The drastic increase in TG levels was prominent (321%), followed by TC (41.5%) and LDL (40%) in HFFD group. In accordance with hypoglycemic property, both K68 and FVF were collectively attenuated hyperlipidemia. All treated groups exhibit decreased (*P* < 0.01) TG levels. Elevated TC and LDL were attenuated by synergetic effect K68 and FVF. Nonetheless, HDL levels were not significantly altered with any of supplement.

The analyzed two adipokines, adiponectin, and leptin levels responded inversely to each other, where adiponectin levels were significantly (*P* < 0.01) decreased and leptin levels were increased with HFFD. Interestingly, decreased adiponectin concentrations were partially restored and increased leptin levels were prominently (*P* < 0.01) inhibited with K68, FVF, and MIX supplements (Table 6).

### 6.6. Influence of K68 and FVF Supplementation on Pro-Inflammatory Cytokines

**Figure 4:**
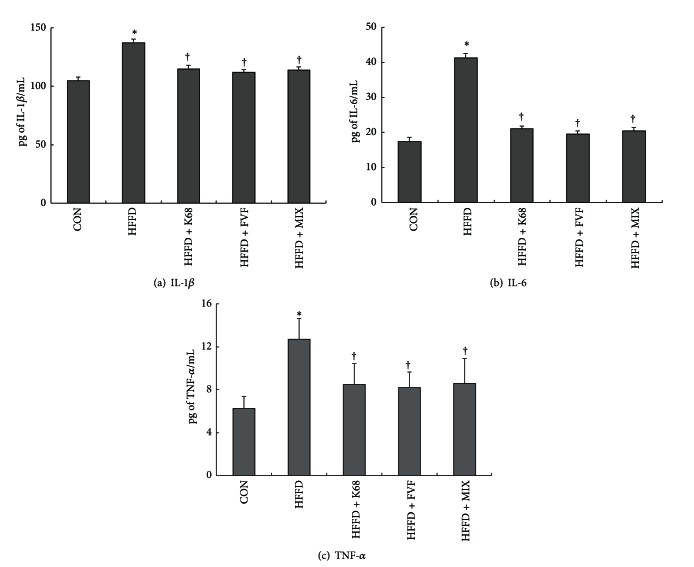
Influence of K68, FVF, and MIX supplementation for 8 weeks on inflammatory cytokines (IL-1*β*, IL-6, and TNF-*α*) in rats fed a HFFD (*N* = 10). *: significant compared to CON; ^†^: significant compared to HFFD group.

HFFD-induced harmful effects on circulating inflammatory cytokines were clearly evidenced by elevated (*P* < 0.01) serum pro-inflammatory cytokines, including IL-1*β*, IL-6, and TNF-*α* concentrations. Among three pro-inflammatory mediators, the levels of IL-6 and TNF-*α* were predominantly increased with HFFD as they reached 137% and 105% elevations, respectively, more than the control diet rats. Another key finding of this study is that elevated inflammatory cytokines were inhibited by K68, FVF, and MIX supplements. The mean values of IL-1*β*, IL-6, and TNF-*α* in supplemented groups were almost similar to the control group. In particular, decreased IL-6 concentration with K68, FVF and MIX treatments was ~50% compared to untreated HFFD group (Figures 4(a), 4(b), and 4(c)).

### 6.7. Beneficial Effect of K68 and FVF on Antioxidant Enzyme Activities

**Figure 5:**
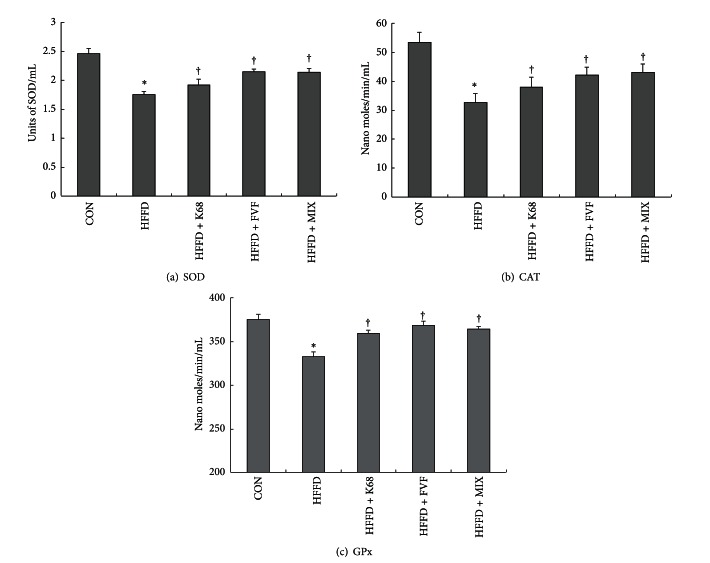
Beneficial effects of K68, FVF, and MIX supplementation for 8 weeks on antioxidant enzyme activities (SOD, CAT, and GPx) in rats fed a HFFD (*N* = 10). *: significant compared to CON; ^†^: significant compared to HFFD group.

To demonstrate whether K68 and FVF and/or combination of both supplements could improve the antioxidant status against hyperglycemic, SOD, CAT, and GPx activities were measured in serum samples. Similar to many other studies, we found a significant (*P* < 0.01) drop in SOD, CAT, and GPx activities in HFFD group. However, decreased antioxidant enzyme activities were significantly (*P* < 0.05) restored with additional supplementation of K68, FVF, and MIX. This data indicates that chronic oral administration of K68, FVF, and MIX are able to cope the oxidative stress caused by HFFD (Figures 5(a), 5(b), and 5(c)).

The antioxidant property of FVF was confirmed by free radical scavenging activity, which was achieved by *in vitro* studies. *In vitro* data convincingly demonstrated that FVF is able to scavenge the free radicals, including DPPH and O_2_^•−^ anion. DPPH scavenging activity was found to be 81% and O_2_^•−^ scavenging activity was indicated as 0.81 IU/200 mg of FVF. Furthermore, FVF possesses ACE inhibitory activity (40%). The total phenol present in the FVF was recorded as 92 mg/200 mg (Table 3).

## 7. Discussion

In this study, we fed a high fat-fructose diet (HFFD) to rats to induce a rat model of insulin resistance. Chronic HFFD apparently showed the basic phenotypes of metabolic syndrome, including obesity, hyperglycemia, hyperinsulinemia, and hyperlipidemia. Evidences from our study clearly demonstrated that supplementation of *L. plantarum* K68 and FVF along with HFFD significantly attenuated the obesity, hyperglycemia, hyperinsulinemia, and hyperlipidemia. For the first time, here we provided experimental evidences for antidiabetic properties of K68 and FVF. Concomitantly, increased serum pro-inflammatory cytokines, IL-1*β*, IL-6, and TNF-*α*, were significantly alleviated by K68, FVF, and MIX supplements, which indicate the potent anti-inflammatory effect. Furthermore, ruined antioxidant system as shown by decreased SOD, CAT, and GPx activities was restored to normal levels, and this restoration was pronounced with MIX. *In vitro* studies further confirmed the antioxidant efficacies of FVF as it showed the DPPH and O_2_^•−^ radical scavenging activities.

It is well known that obesity may influence overall blood parameters, which are responsible for metabolic syndrome. Chronic high fat and fructose consumption, in terms of increased energy intake, has been shown a greater increase in bodyweights [23, 24]. Enhanced fat deposition with high fat-fructose diet may result in weight gain over a period of time. This phenomenon was evidenced by increased epididymal fat weight in HFFD group, which can be considered as a risk factor for cardiovascular disease (CVD). A human study suggested that adolescent overweight will increase CVD among future young and middle-age adults [25]. In this context, controlled bodyweight by *L. plantarum *K68, FVF, and MIX supplements implies the reduced risk of CVD. Soypro, a fermented soymilk with lactic acid bacteria, isolated from Kimchi, was showed to inhibit the expression of transcription factors of adipocyte differentiation [23]. Yadav and colleagues [12] showed that dahi (curd), which contained lactobacillus bacteria prevent the bodyweight gain against high fructose diet. The decreased bodyweight with supplements might be associated with the hypolipidemic property and/or altered adiponectin and leptin concentrations in this study.

Intake of chronic high fat-fructose diet confirmed the IR in rats as we found greater serum insulin and blood glucose levels. Insulin plays a unique role in regulating blood glucose levels and fat metabolism. Recent studies showed that rodents fed a high fat and/or fructose diet for 8 weeks increased the levels of insulin, glucose, and HOMA-IR values that were preceded to IR [24, 26]. Higher insulin levels in fructose group may impair *β*-cells function, since the cells could not cope with the increased insulin demand due to insulin resistance [7]. Impaired insulin homeostasis or glucose tolerance with HFFD is an important predictor of type 2 diabetes, which was reflected by increased risk factors, including HbA1c and HOMA-IR. Decreased insulin sensitivity in type 2 diabetes either by *β*-cells dysfunction or by obesity may affect the circulating lipids [2]. Furthermore, hyperglycemia triggers the ROS production and inflammatory cytokines that are closely associated with CVD [27]. Supplementation of K68 and FVF along with HFFD reversed the hyperglycemia and hyperinsulinemia along with HbA1c and HOMA-IR mean values. These results confirmed that lactobacillus bacteria and FVF products synergistically play a key role in delaying the onset of type 2 diabetes. We assumed that supplements may improve the *β*-cell function. A fermented milk product known as dahi, contained *L. acidophilus *and *L. casei*, was shown to delay the progression of high fructose-induced hyperglycemia, hyperinsulinemia, dyslipidemia, and oxidative stress in rats [12]. Another study showed that oral administration of *L. casei* significantly decreased the blood glucose in KK-Ay mice [28]. Similarly, Tabuchi et al. [11] reported improved glucose tolerance in *L. rhamnosus *GG treated streptozotocin-induced diabetic rats. Antidiabetic properties of bacteria and fermented fruit-vegetable products further suggest the cardioprotective properties against metabolic syndrome.

On the other hand, it has been shown that adipose tissue participates in regulation of bodyweight, glucose, and lipid metabolism via number of secreted proteins, including adiponectin and leptin [29]. Adiponectin exerts a potent insulin-sensitizing effect, activates the glucose uptake, protects against insulin resistance, and acts as anti-inflammatory protein [30, 31]. In this study, decreased adiponectin with HFFD confirmed the impaired insulin sensitivity. However, K68, FVF, and MIX treatments restored the adiponectin concentrations. This data indicates that augmented adiponectin levels may play a vital role in improving the insulin sensitivity and decrease obesity-mediated metabolic complications. Elevated leptin concentrations with high fat-fructose diet were attenuated by K68, FVF, and MIX treatments. In a human study, Naruszewicz et al. [13] reported significantly reduced blood leptin concentrations in smokers after *L. plantarum* 299v contained drink intake.

The important CVD risk factor in type 2 diabetes is dyslipidemia, which is characterized by elevated plasma triglycerides and LDL [32, 33]. Elevated serum TC, TG, and LDL levels with high fat-fructose diet in this study reflect the preoccurrence of CVD. Attenuated dyslipidemia through the synergetic effect of supplements suggests that functional food could be useful to prevent the CVD. Previous studies showed that supplementation of diet with functional food product containing fruit juice, fermented oat, and *L. plantarum *significantly lowers the LDL concentrations in patients with moderately higher cholesterol levels [34]. Another study reported that serum TC levels were significantly decreased in rats fed fermented milk with both* L. casei *and* Streptococcus thermophilus *TMC 1543, while TG levels were decreased only with fermented milk [35]. The hypolipidemic activity of functional foods may be due to the specific action on cholesterol metabolizing enzymes in liver, promotion of cholesterol excretion though feces, and inhibition of cholesterol absorption by binding of cholesterol to LAB cells [36–38].

HFFD-induced altered lipid profiles and hyperglycemia are closely associated with elevated pro-inflammatory cytokines, as we recorded increased concentrations of IL-1*β*, IL-6, and TNF-*α*. Increased pro-inflammatory mediators can tip the crucial balance between pro- and anti-inflammatory mediators, thus results in inflammation and influences the normal physiological functions. TNF-*α*, contributing to development of IR, has been shown to elevate in obese rodents [39]. Our findings are in agreement with previous results, which showed the increased pro-inflammatory cytokines with high fat and/or fructose diets in rodents [24, 40]. The major therapeutic applications of K68 and FVF supplements were evidenced by decreasing all pro-inflammatory cytokines. Recently, we reported that production of pro-inflammatory cytokines, IL-1*β*, IL-6, and TNF-*α*, was significantly decreased by oral administration of *L. plantarum *K68 in DSS-induced ulcerative colitis mice. Increased mRNA expression of TNF-*α* in DSS group was also found to lower in K68 treated groups [16]. Chon et al. [41] showed that ultrafiltrates of *L. plantarum* KFCC11689P metabolic products inhibit IL-6 and TNF-*α* production in lipopolysaccharide (LPS)-induced RAW 264.7 cells. Since elevated IL-1*β*, IL-6, and TNF-*α* were referred to as independent predictors of diabetes [42], decreased concentrations reveal the antidiabetic properties of functional food. The combined effect of K68 and FVF as anti-inflammatory substances may play a key role to attenuate the inflammation. However, the detailed mechanism behind this anti-inflammatory activity needs further investigations.

Hyperglycemia-induced negative impact on antioxidant status was revealed by decreased antioxidant enzyme activities. Emerging evidences show that chronic fat or fructose intake triggers the reactive oxygen species (ROS) production, including superoxide radicals (O_2_^•−^) that mediate insulin resistance [5, 43]. Typically, O_2_^•−^ radicals scavenge into hydrogen peroxide (H_2_O_2_) by SOD, and then H_2_O_2_ rapidly converted into water and oxygen by CAT and GPx enzymes in a site-specific manner. Decrease in these enzyme activities results in accumulation of ROS and therefore causes oxidative stress. Rise in O_2_^•−^ production and/or glycation of active sites of SOD under hyperglycemic condition may be the possible reason for SOD reduction [6, 44]. Similar to our studies, Francini et al. [7] reported decreased CAT activity with 10% fructose diet for 3 weeks, and Kannappan et al. [6] found reduced GPx activity in rats fed with 60% fructose for 60 days. K68 and FVF supplements were found to restore the SOD, CAT, and GPx activities. Since accumulation of O_2_^•−^ plays a key role in the progression oxidative stress, normalizing the O_2_^•−^ production may prevent the hyperglycemic mediated oxidative stress [45]. Improved antioxidant enzyme activities with supplements may effectively eradicate the excessive ROS and thereby prevent the oxidative stress. Studies have indicated that *L. plantarum* encodes genes for various oxidative stress-related protein such as catalase, thiol reductase, and GPx, while it is not encoded with SOD genes [46, 47]. On the other hand, antioxidants, including vitamin E and selenium that are present in FVF, may be responsible for its antioxidant activity.

## 8. Conclusion

For the first time, our study demonstrated that *L. plantarum *K68 and fermented fruit-vegetables supplements contribute to decrease the hyperglycemia, hyperinsulinemia, and hyperlipidemia in HFFD-fed rats. The therapeutic effects may attribute to increased antioxidant status and decreased pro-inflammatory cytokines. These findings conclude that specific LAB, *L. plantarum *K68, isolated from Taiwanese traditional food *fu-tsai*, and FVF products synergistically exert promising antidiabetic, anti-inflammatory, and antioxidant properties. Our results suggest that inclusion of K68 and FVF to the Western style diet may decrease the risk of diabetes. Due to their effective medicinal properties, both K68 and FVF can be considered as alternative medicine to prevent insulin resistance-associated metabolic disorders.

## Figures and Tables

**Table 1:** Composition of standard and experimental diets.

AIN-93M	Standard diet (w/w)	High fat-fructose diet (w/w)
Corn starch	46%	46%
Dextrin	15.5%	15.5%
Casein vitamin free	14%	14%
Sucrose	10%	28%
Fructose	—	20%
Powdered cellulose	5%	5%
Soybean oil	4%	4% + 18% (lard)
AIN 93M mineral mix	3.5%	3.5%
AIN 93 vitamin mix	1%	1%
Choline bitartrate	0.25%	0.25%
L-Cystine	0.18%	0.18%
t-Butylhydroquinone	0.0008%	0.0008%
Energy (Kcal/30 g/day)	114	141

**Table 2:** Quantified bioactive compounds in fruit-vegetable ferment.

Name of the component	Content/100 g
Crude fat	0.48 g
Crude protein	2.12 g
Total carbohydrate	148.97 g
Dietary fiber	2.70 g
Sodium (Na)	87.44 mg
Potassium (K)	490.04 mg
Calcium (Ca)	48.43 mg
Magnesium (Mg)	39.4 mg
Ferrous (Fe)	1.35 mg
Phosphorous (P)	61.08 mg
Vitamine B-1	0.03 mg
Vitamin B-2	0.16 mg
Vitamin B-6	0.32 mg
Vitamin E	0.10 mg
Pantothenic acid	0.29 mg
Folic acid	34.14 mg
Choline	14.55 mg
Inositol	100.77 mg
Biotin	9.15 *μ*g

All the values are expressed with respective units per 100 g of FVF.

**Table 3:** Existence of antioxidant compounds and free radical scavenging properties (*in vitro*) of FVF.

Antioxidant compounds/capacity	Units
Vitamin E	0.10 mg/100 mg
Selenium	0.06 ppm/100 mg
DPPH scavenging ability, 100% (Vit C 1 mg/mL)	81%/200 mg
Superoxide radical scavenging activity	0.81 IU/200 mg
Total phenols	92 mg/200 mg
ACE inhibition	40%/200 mg

**Table 4:** Bodyweight gain over a period of 8 weeks and the final liver, kidney, and epididymal fat weights in HFFD, K68, FVF, and MIX supplemented groups. All the values are expressed in grams for 10 replicates (*N* = 10).

Parameters	CON	HFFD	HFFD + K68	HFFD + FVF	HFFD + MIX
Bodyweight gain (g) (final-initial weight)	288.7 ± 1.6	341.6 ± 5.73*	302.5 ± 2.4^∗†^	316 ± 5.8^∗†^	315.62 ± 4^∗†^
Liver (g)	2.49 ± 0.10	2.79 ± 0.05*	2.33 ± 0.13^†^	2.49 ± 0.08^†^	2.61 ± 0.09
Kidney (g)	0.50 ± 0.01	0.54 ± 0.02	0.54 ± 0.02	0.53 ± 0.01	0.55 ± 0.01
Epididymal fat (g)	2.06 ± 0.08	2.48 ± 0.03*	2.35 ± 0.06	2.26 ± 0.06	2.13 ± 0.03^†^
Average food intake (g/day)	28.4 ± 0.6	29.5 ± 0.2	27.5 ± 0.6	27.3 ± 0.4	27.3 ± 0.6
FCE ratio	0.097 ± 0.002	0.087 ± 0.003*	0.09 ± 0.002	0.087 ± 0.002*	0.087 ± 0.002*

*Significant compared to CON group.

^†^Significant compared to HFFD group.

Food conversion efficiency (FCE) ratio = food intake (g)/weight gain (kg) × 10^2^.

**Table 5:** Changes in blood glucose, insulin, HbA1c, and HOMA-IR values over a period of HFFD feeding and effect of K68, FVF, and MIX supplementation (*N* = 10).

Parameters	CON	HFFD	HFFD + K68	HFFD + FVF	HFFD + MIX
Week 6					
Glucose (mg/dL)	93.6 ± 3.0	144.4 ± 2.8*	138.4 ± 3.2*	133.8 ± 2.6^∗†^	128.1 ± 2.4^∗†#^
Insulin (ng/mL)	1.27 ± 0.03	2.05 ± 0.04*	1.89 ± 0.03*	1.77 ± 0.02^∗†^	1.58 ± 0.03^∗†#^
HOMA-IR	1.32 ± 0.04	3.28 ± 0.08*	2.92 ± 0.10^∗†^	2.63 ± 0.05^∗†^	2.26 ± 0.07^∗†#^

Week 8					
Glucose (mg/dL)	95.1 ± 2.7	165 ± 2.0*	152.5 ± 3.5^∗†^	140.4 ± 3.6^∗†^	137.1 ± 2.3^∗†#^
Insulin (ng/mL)	1.3 ± 0.03	2.74 ± 0.05*	2.3 ± 0.04^∗†^	2.29 ± 0.04^∗†^	2.13 ± 0.03^∗†^
HbA1c (%)	4.8 ± 0.2	5.8 ± 0.2*	5.4 ± 0.2*	5.2 ± 0.2^†^	5.1 ± 0.2^†^
HOMA-IR	1.39 ± 0.05	5.08 ± 0.09*	3.94 ± 0.12^∗†^	3.62 ± 0.15^∗†^	3.27 ± 0.08^∗†#^

*Significant compared to CON group.

^†^Significant compared to HFFD group.

^#^Significant compared to K68 and FVF groups.

**Table 6:** Effect of K68, FVF, and MIX supplementation on altered lipid profile (TC, TG, HDL, and LDL) and adipokines (adiponectin and leptin) in HFFD-induced IR rats (*N* = 10).

Parameters	CON	HFFD	HFFD + K68	HFFD + FVF	HFFD + MIX
TC (mg/dL)	68.9 ± 1.1	97.5 ± 2.2*	93.5 ± 2.6*	92.6 ± 1.7*	86.8 ± 1.9^∗†#^
TG (mg/dL)	87.8 ± 1.9	203.6 ± 2*	181.5 ± 1.6^∗†^	183.5 ± 1.5^∗†^	181.5 ± 1.6^∗†^
LDL (mg/dL)	15.3 ± 1.0	21.3 ± 2.5*	21.4 ± 2.8*	21 ± 1.7*	15.3 ± 1.7^†#^
HDL (mg/dL)	36.1 ± 0.5	35.5 ± 0.4	35.8 ± 0.3	34.9 ± 0.7	35.2 ± 0.4
Adiponectin (ng/mL)	44.5 ± 0.7	32.1 ± 0.7*	38.4 ± 0.9^†^	37.8 ± 1.1^∗†^	39.2 ± 0.8^†^
Leptin (pg/mL)	39.8 ± 1	68.1 ± 1.1*	48 ± 1^∗†^	43.8 ± 0.8^∗†^	49.5 ± 1^∗†^

*Significant compared to CON group.

^†^Significant compared to HFFD group.

^#^Significant compared to K68 and FVF groups.

## References

[1] Miranda PJ, DeFronzo RA, Califf RM, Guyton JR (2005). Metabolic syndrome: definition, pathophysiology, and mechanisms. *American Heart Journal*.

[2] Elliott SS, Keim NL, Stern JS, Teff K, Havel PJ (2002). Fructose, weight gain, and the insulin resistance syndrome. *American Journal of Clinical Nutrition*.

[3] Potenza MV, Mechanick JI (2009). The metabolic syndrome: definition, global impact, and pathophysiology. *Nutrition in Clinical Practice*.

[4] Odermatt A (2011). The Western-style diet: a major risk factor for impaired kidney function and chronic kidney disease. *American Journal of Physiology—Renal Physiology*.

[5] Matsuzawa-Nagata N, Takamura T, Ando H (2008). Increased oxidative stress precedes the onset of high-fat diet-induced insulin resistance and obesity. *Metabolism*.

[6] Kannappan S, Palanisamy N, Anuradha CV (2010). Suppression of hepatic oxidative events and regulation of eNOS expression in the liver by naringenin in fructose-administered rats. *European Journal of Pharmacology*.

[7] Francini F, Castro MC, Schinella G (2010). Changes induced by a fructose-rich diet on hepatic metabolism and the antioxidant system. *Life Sciences*.

[8] Battcock M, Azam-Ali S (1998). *Fermented Fruits and Vegetables: A Globsl Perspective*.

[9] De Vos WM, Hugenholtz J (2004). Engineering metabolic highways in Lactococci and other lactic acid bacteria. *Trends in Biotechnology*.

[10] Kalliomäki M, Salminen S, Arvilommi H, Kero P, Koskinen P, Isolauri E (2001). Probiotics in primary prevention of atopic disease: a randomised placebo-controlled trial. *Lancet*.

[11] Tabuchi M, Ozaki M, Tamura A (2003). Antidiabetic effect of *Lactobacillus* GG in streptozotocin-induced diabetic rats. *Bioscience, Biotechnology and Biochemistry*.

[12] Yadav H, Jain S, Sinha PR (2007). Antidiabetic effect of probiotic dahi containing *Lactobacillus acidophilus* and *Lactobacillus casei* in high fructose fed rats. *Nutrition*.

[13] Naruszewicz M, Johansson ML, Zapolska-Downar D, Bukowska H (2002). Effect of *Lactobacillus plantarum* 299v on cardiovascular disease risk factors in smokers. *American Journal of Clinical Nutrition*.

[14] Chao SH, Tomii Y, Watanabe K, Tsai YC (2008). Diversity of lactic acid bacteria in fermented brines used to make stinky tofu. *International Journal of Food Microbiology*.

[15] Chao SH, Wu RJ, Watanabe K, Tsai YC (2009). Diversity of lactic acid bacteria in suan-tsai and fu-tsai, traditional fermented mustard products of Taiwan. *International Journal of Food Microbiology*.

[16] Liu YW, Su YW, Ong WK, Cheng TH, Tsai YC (2011). Oral administration of *Lactobacillus plantarum* K68 ameliorates DSS-induced ulcerative colitis in BALB/c mice via the anti-inflammatory and immunomodulatory activities. *International Immunopharmacology*.

[17] Fröhlich RH, Kunze M, Kiefer I (1997). Cancer preventive impact of naturally occurring, non-nutritive constituents in food. *Acta Medica Austriaca*.

[18] FDA U Guidance for Industry, Estimating the maximum safe starting dose in initial clinical trials for therapeutics in adult healthy volunteers. http://www.fda.gov/cder/guidance/index.htm.

[19] Friedewald WT, Levy RI, Fredrickson DS (1972). Estimation of the concentration of low-density lipoprotein cholesterol in plasma, without use of the preparative ultracentrifuge. *Clinical Chemistry*.

[20] Liyana-Pathirana CM, Shahidi F (2005). Antioxidant activity of commercial soft and hard wheat (Triticum aestivum L.) as affected by gastric pH conditions. *Journal of Agricultural and Food Chemistry*.

[21] Robak J, Gryglewski RJ (1988). Flavonoids are scavengers of superoxide anions. *Biochemical Pharmacology*.

[22] Wolfe K, Wu X, Liu RH (2003). Antioxidant activity of apple peels. *Journal of Agricultural and Food Chemistry*.

[23] Kim NH, Moon PD, Kim SJ (2008). Lipid profile lowering effect of Soypro*™* fermented with lactic acid bacteria isolated from Kimchi in high-fat diet-induced obese rats. *BioFactors*.

[24] Wada T, Kenmochi H, Miyashita Y (2010). Spironolactone improves glucose and lipid metabolism by ameliorating hepatic steatosis and inflammation and suppressing enhanced gluconeogenesis induced by high-fat and high-fructose diet. *Endocrinology*.

[25] Bibbins-Domingo K, Coxson P, Pletcher MJ, Lightwood J, Goldman L (2007). Adolescent overweight and future adult coronary heart disease. *The New England Journal of Medicine*.

[26] Suwannaphet W, Meeprom A, Yibchok-Anun S, Adisakwattana S (2010). Preventive effect of grape seed extract against high-fructose diet-induced insulin resistance and oxidative stress in rats. *Food and Chemical Toxicology*.

[27] Ceriello A, Testa R (2009). Antioxidant anti-inflammatory treatment in type 2 diabetes. *Diabetes Care*.

[28] Matsuzaki T, Yamazaki R, Hashimoto S, Yokokura T (1997). Antidiabetic effects of an oral administration of *Lactobacillus casei* in a non-insulin-dependent diabetes mellitus (NIDDM) model using KK-A(y) mice. *Endocrine Journal*.

[29] Athyros VG, Tziomalos K, Karagiannis A, Anagnostis P, Mikhailidis DP (2010). Should adipokines be considered in the choice of the treatment of obesity-related health problems?. *Current Drug Targets*.

[30] Kadowaki T, Yamauchi T, Kubota N, Hara K, Ueki K, Tobe K (2006). Adiponectin and adiponectin receptors in insulin resistance, diabetes, and the metabolic syndrome. *Journal of Clinical Investigation*.

[31] Kubota N, Terauchi Y, Yamauchi T (2002). Disruption of adiponectin causes insulin resistance and neointimal formation. *Journal of Biological Chemistry*.

[32] Reifel-Miller A, Otto K, Hawkins E (2005). A peroxisome proliferator-activated receptor *α*/*γ* dual agonist with a unique *in vitro* profile and potent glucose and lipid effects in rodent models of type 2 diabetes and dyslipidemia. *Molecular Endocrinology*.

[33] Mooradian AD (2009). Dyslipidemia in type 2 diabetes mellitus. *Nature Clinical Practice Endocrinology and Metabolism*.

[34] Bukowska H, Pieczul-Mroz J, Jastrzebska M, Chelstowski K, Naruszewicz M (1998). Decrease in fibrinogen and LDL-cholesterol levels upon supplementation of diet with *Lactobacillus plantarum* in subjects with moderately elevated cholesterol. *Atherosclerosis*.

[35] Kawase M, Hashimoto H, Hosoda M, Morita H, Hosono A (2000). Effect of administration of fermented milk containing whey protein concentrate to rats and healthy men on serum lipids and blood pressure. *Journal of Dairy Science*.

[36] Fukushima M, Nakano M (1996). Effects of a mixture of organisms, *Lactobacillus acidophilus* or *Streptococcus faecalis* on cholesterol metabolism in rats fed on a fat- and cholesterol-enriched diet. *British Journal of Nutrition*.

[37] Hashimoto H, Yamazaki K, He F, Kawase M, Hosoda M, Hosono A (1999). Hypocholesterolemic effects of *Lactobacillus casei* subsp, casei TMC, 0409 strain observed in rats fed cholesterol contained diets. *Animal Science Journal*.

[38] Rao DR, Chawan C, Pulusani S (1981). Influence of milk and thermophilus milk on plasma cholesterol levels and hepatic cholesterogenesis in rats. *Journal of Food Science*.

[39] Hotamisligil GS, Shargill NS, Spiegelman BM (1993). Adipose expression of tumor necrosis factor-*α*: direct role in obesity-linked insulin resistance. *Science*.

[40] Cani PD, Amar J, Iglesias MA (2007). Metabolic endotoxemia initiates obesity and insulin resistance. *Diabetes*.

[41] Chon H, Choi B, Lee E, Lee S, Jeong G (2009). Immunomodulatory effects of specific bacterial components of *Lactobacillus plantarum* KFCC11389P on the murine macrophage cell line RAW 264*·*7. *Journal of Applied Microbiology*.

[42] Kern PA, Di Gregorio GB, Lu T, Rassouli N, Ranganathan G (2003). Adiponectin expression from human adipose tissue: relation to obesity, insulin resistance, and tumor necrosis factor-*α* expression. *Diabetes*.

[43] Houstis N, Rosen ED, Lander ES (2006). Reactive oxygen species have a causal role in multiple forms of insulin resistance. *Nature*.

[44] Oda A, Bannai C, Yamaoka T, Katori T, Matsushima T, Yamashita K (1994). Inactivation of Cu,Zn-superoxide dismutase by *in vitro* glycosylation and in erythrocytes of diabetic patients. *Hormone and Metabolic Research*.

[45] Nishikawa T, Edelstein D, Du XL (2000). Normalizing mitochondrial superoxide production blocks three pathways of hyperglycaemic damage. *Nature*.

[46] Kleerebezem M, Boekhorst J, Van Kranenburg R (2003). Complete genome sequence of *Lactobacillus plantarum* WCFS1. *Proceedings of the National Academy of Sciences of the United States of America*.

[47] Archibald FS, Fridovich I (1981). Manganese and defenses against oxygen toxicity in *Lactobacillus plantarum*. *Journal of Bacteriology*.

